# Quantitative Evaluation of White Matter Injury by Cranial Ultrasound to Detect the Effects of Parenteral Nutrition in Preterm Babies: An Observational Study

**DOI:** 10.3390/jimaging10090224

**Published:** 2024-09-10

**Authors:** Gianluigi Laccetta, Maria Chiara De Nardo, Raffaella Cellitti, Maria Di Chiara, Monica Tagliabracci, Pasquale Parisi, Flavia Gloria, Giuseppe Rizzo, Alberto Spalice, Gianluca Terrin

**Affiliations:** 1 Department of Maternal Infantile and Urological Sciences, Sapienza University of Rome, 00185 Rome, Italy; mariachiara.denardo@uniroma1.it (M.C.D.N.); rafcell@hotmail.it (R.C.); maria.dichiara@uniroma1.it (M.D.C.); monicatagliabracci@gmail.com (M.T.); gloria.1891102@studenti.uniroma1.it (F.G.); giuseppe.rizzo@uniroma1.it (G.R.); alberto.spalice@uniroma1.it (A.S.); gianluca.terrin@uniroma1.it (G.T.); 2 Department of Neuroscience, Mental Health and Sense Organs (NESMOS), Faculty of Medicine and Psychology, Sant’Andrea University Hospital, Sapienza University of Rome, 00185 Rome, Italy; pasquale.parisi@uniroma1.it

**Keywords:** white matter, echogenicity, pixel brightness intensity, cranial ultrasound, enteral nutrition, parenteral nutrition, preterm newborn

## Abstract

Nutrition in early life has an impact on white matter (WM) development in preterm-born babies. Quantitative analysis of pixel brightness intensity (PBI) on cranial ultrasound (CUS) scans has shown a great potential in the evaluation of periventricular WM echogenicity in preterm newborns. We aimed to investigate the employment of this technique to objectively verify the effects of parenteral nutrition (PN) on periventricular WM damage in preterm infants. Prospective observational study including newborns with gestational age at birth ≤32 weeks and/or birth weight ≤1500 g who underwent CUS examination at term-equivalent age. The echogenicity of parieto–occipital periventricular WM relative to that of homolateral choroid plexus (RE_CP_) was calculated on parasagittal scans by means of quantitative analysis of PBI. Its relationship with nutrient intake through enteral and parenteral routes in the first postnatal week was evaluated. The study included 42 neonates for analysis. We demonstrated that energy and protein intake administered through the parenteral route positively correlated with both right and left RE_CP_ values (parenteral energy intake vs. right RE_CP_: r = 0.413, *p* = 0.007; parenteral energy intake vs. left RE_CP_: r = 0.422, *p* = 0.005; parenteral amino acid intake vs. right RE_CP_: r = 0.438, *p* = 0.004; parenteral amino acid intake vs. left RE_CP_: r = 0.446, *p* = 0.003). Multivariate linear regression analysis confirmed these findings. Quantitative assessment of PBI could be considered a simple, risk-free, and repeatable method to investigate the effects of PN on WM development in preterm neonates.

## 1. Introduction

The deleterious impact of enhanced parenteral nutrition (PN) on brain development still represents an emerging body of evidence [1]. Although prior research suggested that children receiving a nutrient-enriched diet by the enteral route in postnatal weeks after preterm birth had significantly larger encephalic volumes and better neurodevelopment [2], more recent investigations showed that higher macronutrient intakes through the parenteral route early in life resulted in worse neurological outcome [1,3]. The reasons for such divergent results could lie in the route of administration of nutrients: according to this hypothesis, higher nutrient intakes through the enteral route could be associated with a reduced risk of brain damage; conversely, parenteral administration of large amounts of the same substances could have a negative impact on neurological outcomes in preterm-born babies. White matter (WM) is highly susceptible to damage in these patients; indeed, WM injury is detected to some extent in up to 50% of very low birth weight (VLBW) infants [4,5,6,7,8,9]. Prevention of even milder forms of WM injury is crucial, considering their relationship with long-term neurodevelopmental impairment [4,6,10].

Given the influence of nutritional strategy on cerebral development, defining the effects of different dietary approaches on brain injury is a priority for neonatologists. Unfortunately, the diagnostic method to identify WM injury associated with nutrient intake is largely undefined. Various techniques are currently used to investigate brain injury in preterm-born infants, including magnetic resonance imaging (MRI) that is deemed as the gold-standard approach in this sector [4,5]. However, this method has some limitations, as it is costly and involves both transfer and sedation of the baby [4,5,11], which could increase parental stress [12]. Furthermore, accessibility to MRI is often limited, which renders serial scanning difficult [4,5]. In contrast, cranial ultrasound (CUS) is a bedside technology that permits secure and trustworthy serial imaging, even in unstable patients; it also allows the diagnosis of a broad range of cerebral pathological conditions and the evaluation of damage over time, as well as brain development [4,5,13,14,15]. However, CUS is limited by potential discrepancies in technical acquisition of images, and intra- and interobserver variability, which burden the analysis of scans [4,16,17]. To limit these aspects, especially the problem of subjective interpretation of ultrasonography, different techniques have been developed in the last decades, including the employment of nanotechnology [4,18,19,20,21,22,23,24,25,26,27,28,29]. Among these tools, quantitative analysis of pixel brightness intensity (PBI) [30] has been successfully used to measure the echogenicity of periventricular WM and to assess the relationship between computed values and short- and middle-term neurodevelopment in preterm newborns [4,18]. Starting from these premises, we aimed to investigate the employment of quantitative evaluation of PBI to objectively verify the effects of PN on periventricular WM damage in preterm babies.

## 2. Materials and Methods

### 2.1. Patients

In a prospective observational investigation, we recruited preterm newborns with gestational age (GA) at birth ≤32 weeks and/or birth weight (BW) ≤1500 g, consecutively admitted to the level III Neonatal Intensive Care Unit (NICU) [31] of Policlinico Umberto I Hospital, Sapienza University of Rome, between 1 January and 31 December 2023. Infants were excluded if they had WM injury ≥ grade 2 according to de Vries et al. [32], subcortical WM damage, grade III or IV intraventricular hemorrhage (IVH), hydrocephalus, porencephaly, major congenital malformations, epileptic encephalopathies [33], hereditary metabolic diseases, perinatal asphyxia, or cerebral infections. Genetic syndromes represented an additional exclusion criterion; the mothers of all eligible infants had to exhibit a negative result at the non-invasive prenatal testing (NIPT) before enrollment. Accordingly, patients would have been excluded even in the case of postnatal diagnosis of a genetic disorder of any kind. Inclusion and exclusion criteria were presented to all subjects belonging to the medical and nursing staff of our NICU during dedicated meetings, which were performed before the beginning of the enrollment process. Thus, physicians were unaware about the study aims but responsible for the enrollment of eligible patients.

### 2.2. Nutritional Protocol

In stable or relatively stable newborns, we initiated enteral nutrition (EN) as soon as possible after birth and, in all cases, within 48 h after birth [1]. In unstable patients, EN was started as soon as clinical conditions improved [1]. In this case, it was possible to commence EN even after the first 48 h of life [1]. In all cases, EN was initiated as minimal enteral feeding (10–20 mL/kg/day) [1]. Thus, the amount of EN was increased by 20–30 mL/kg every day, in relation to the degree of tolerance of EN. We preferred to feed enrolled babies by means of maternal milk. Thus, if available, fresh maternal milk without fortifications was administered through the enteral route. In case maternal milk was not available or sufficient, proper preterm formula was given; donor breast milk was not available during the study period. We considered the following signs as markers of feeding intolerance: vomiting, severe abdominal distension associated with ileus and visible intestinal loops, blood in the stools, and even some systemic disorders (i.e., apnea, bradycardia, inadequate perfusion, hemodynamic instability, and lethargy). In cases when one or more of the previous signs were observed, EN was withheld for at least 24 h [34,35]. PN was administered from birth to ensure proper fluid, electrolyte, and nutrient intake before exclusive enteral feeding (100–120 kcal/kg/day) was achieved. The overall fluid intake administered with EN and PN started with 80 mL/kg/day and slowly increased by 10–20 mL/kg/day until reaching 150 mL/kg/day. Our protocol foresaw parenteral administration of 2.5 g of amino acids on the first day of life; thus, we increased overall amino acid intake up to 3.2 g/kg/day, with ≥25 kcal for each gram of amino acids. Total glucose intake was started at 6 g/kg/day and increased up to 13 g/kg/day. Overall lipid intake was started at 1 g/kg/day and increased up to 3.5 g/kg/day. In daily clinical practice, fluid and nutrient intakes through minimal enteral feeding were not accounted in the calculation of overall daily intakes. Since EN exceeded 20 mL/kg/day, target doses of macronutrients referred to enteral plus parenteral intakes of each one; particularly, we adjusted parenteral intake of these substances based on the amount of EN tolerated. Parenteral and enteral intakes of energy and macronutrients in the first week of life (measured as kcal/kg/first week and g/kg/first week, respectively) expressed the actual amount of energy and macronutrients coming from enteral and parenteral routes of administration over the first 7 days of life. Sources of parenteral macronutrients were as follows: (1) for amino acids: TrophAmine^®^ 6%, Braun Medical Inc., Irvine, CA, USA; (2) For lipids: Smoflipid^®^, Fresenius Kabi, Lake Zurich, IL, USA; (3) For carbohydrates: dextrose injection 10–33%, Fresenius Kabi, Lake Zurich, IL, USA. Preterm human milk was assumed to contain 65 kcal/100 mL (proteins 1.5 g/100 mL, lipids 3.5 g/100 mL, carbohydrates 6.9 g/100 mL). Macronutrient contents of formula milk (Pre-Nidina Nestlè^®^: proteins 2.1 g/100 mL, lipids 3.9 g/100 mL, carbohydrates 7.8 g/100 mL, energy 75 kcal/100 mL) were obtained from the published manufacturer’s labels.

### 2.3. Acquisition and Analysis of CUS Scans

**Figure 1:**
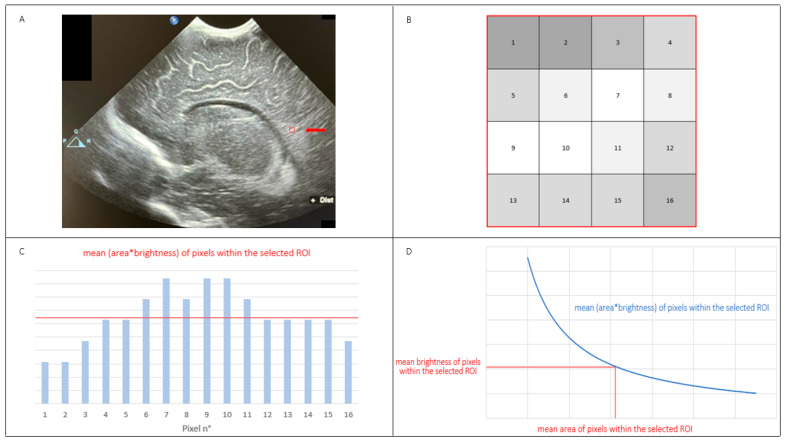
Details about the methodology used to calculate mean pixel brightness intensity (mPBI) values within selected regions of interest (ROIs). Firstly, we delimited a specific ROI; in this case, ROI is located within the parieto-occipital periventricular white matter (red box in **A**, as indicated by the arrow). Based on its size, the selected ROI contains a number of pixels (i.e., the smallest unit of a digital image); each pixel has its own brightness ranging from 0 (black) to 255 (white)–(**B**). The contribution of each pixel in determining the mPBI of the selected ROI depends on its brightness, and even on its location within the ROI; the graph in (**C**) represents this concept. By dividing the sum of (area×brightness) of all pixels for the number of pixels within the ROI, we obtain the mean (area×brightness) of the pixels. If we divide the mean (area×brightness) for the mean surface of pixels, we obtain the mPBI value of pixels within the selected ROI (**D**).

CUS examinations were carried out at term-equivalent age (TEA), or 37^0/7^–41^6/7^ weeks’ postmenstrual age (PMA). Ultrasonographic scans were performed by a single researcher, a neonatologist with 23 years of experience in CUS and neonatal neuroimaging, who was unaware of the study purposes. Particulars of image acquisition have been outlined elsewhere and recorded hereinafter [4]. CUS examinations were done by using an Affiniti 50G scanner (Philips Healthcare, Andover, MA, USA) with an 8–5 MHz convex probe. No attempts were performed to restrict the ultrasound machine or the investigator’s ability to capture the best images possible. Each CUS examination comprised a minimum of six standard coronal and five parasagittal scans through the anterior fontanelle. The sonographer was responsible for the diagnosis of some conditions which constituted a reason for exclusion of patients (e.g., WM injury ≥grade 2 according to de Vries et al. [32], subcortical WM damage, grade III or IV IVH, hydrocephalus, porencephaly, and malformations of the central nervous system). In case of diagnostic doubt, the sonographer met two expert physicians, and together they discussed the case and reached a definitive diagnosis. In the event that a condition representing an ultrasonographic exclusion criterion was diagnosed, the patient was dismissed from the study and included in a dedicated follow-up service. Two investigators, unaware of clinical information, acted as third-party observers. They selected right and left parasagittal scans through the body of the lateral ventricle and carried out quantitative analysis of the echogenicity of parieto-occipital periventricular WM relative to that of homolateral choroid plexus (CP) by means of QLAB13 (Philips, Andover, MA, USA) image processing software. First, the anatomical borders of parieto-occipital periventricular WM and the middle part of homolateral CP were manually drawn on the same parasagittal scans; thus, square-shaped regions of interest (ROIs) with a fixed surface (1.00 mm^2^) in all scans were delineated and positioned on the most hyperechoic portion of both the parieto-occipital periventricular WM and the middle part of the ipsilateral CP. In this way, we automatically obtained the mean PBI (mPBI) value for the selected ROIs; possible values ranged from 0 (black) to 255 (white). The echogenicity of parieto–occipital periventricular WM relative to that of homolateral CP (RE_CP_) corresponded to the ratio between the mPBI value of parieto-occipital periventricular WM and the mPBI value of the middle part of CP (mPBI_WM_/mPBI_CP_) within the selected ROIs. Each observer had five attempts to detect the highest possible RE_CP_ value in both sides of the brain. At this point, every single researcher selected the highest RE_CP_ value he found in each cerebral hemisphere. Thus, we used the highest overall value of RE_CP_ for each side, independently from the researcher, for calculation of mean right and left RE_CP_ values and their standard deviations, and for correlation and multivariate analyses. Consequently, we selected for these purposes the highest of ten RE_CP_ values (five for each researcher) for both sides of the brain. In addition, we considered the highest RE_CP_ value from each researcher for each cerebral hemisphere and calculated the inter-observer variability relative to the measurement of the highest RE_CP_ value in each side of the brain. Several days later, both observers performed the same quantitative analysis of brain echogenicity. Thus, we used the highest right and the highest left RE_CP_ values coming from the two separate analyses by the two researchers over time and calculated the intra-observer variability relative to the measurement of the highest RE_CP_ value in both sides of the brain in the two different time points. CUS scans in which the parieto-occipital periventricular WM was not completely evident were not used for the aims of the present investigation. Details about the technology used to calculate mPBI values within the selected ROIs are provided in Figure 1. 

### 2.4. Statistical Analysis

Statistical Package for Social Science software (SPSS Inc., Chicago, IL, USA), version 27.0, was used to perform the statistical analysis. Normality was checked by means of the Shapiro–Wilk test; mean and standard deviation (SD) summarized continuous variables, while number and percentage described categorical variables. The correlation between RE_CP_ values and continuous variables (including macronutrient and energy intakes by both enteral and parenteral routes) was evaluated by using Pearson correlation. In order to assess the impact of potential confounding variables on the relation between nutritional intakes in the first week of life and RE_CP_ values at TEA, we carried out multivariate linear regression analysis using the statistically significant factors at univariate analysis and potentially crucial postnatal variables as covariates. The intraclass correlation coefficients (ICCs) were calculated to assess the inter- and intra-observer variability in the identification of the highest RE_CP_ values of both sides of the brain, as reported elsewhere [36]. In the assessment of intra-observer reliability, we reported the lowest ICC for each side. For statistical significance, we considered a *p* value < 0.05. The post-hoc statistical power for multiple regression studies was calculated by means of the Free Statistics Calculators version 4.0 (available online at https://www.danielsoper.com/statcalc/, accessed on 25 August 2024). A statistician blinded to the study aims, characteristics of enrolled patients, and results of the quantitative evaluation of WM relative echogenicity, analyzed the encrypted data. Codes were revealed at the end of statistical analysis.

### 2.5. Ethics

The study was performed in accordance with the World Medical Association Declaration of Helsinki for medical research including human subjects. The investigation procedure was validated by the Ethics Committee of Policlinico Umberto I Hospital, Sapienza University of Rome (no. 5089; 13 September 2018). Written informed consent was received from parents or legal guardians of all infants before enrollment.

## 3. Results

Forty-six eligible newborns were identified. We excluded four patients because of cystic periventricular leukomalacia (*n* = 2), congenital infection (*n* = 1), and genetic syndrome (*n* = 1); thus, 42 neonates were included for analysis. The main characteristics of the study population are shown in Table 1 and Table 2. Enteral and parenteral nutritional intakes of enrolled infants in the first week of life are reported in Table 3. Mean right and left RE_CP_ values at TEA were 0.83 ± 0.12 and 0.79 ± 0.12, respectively; these values are similar to those previously found in a population of newborns with GA at birth < 32 weeks [4].

**Figure 2:**
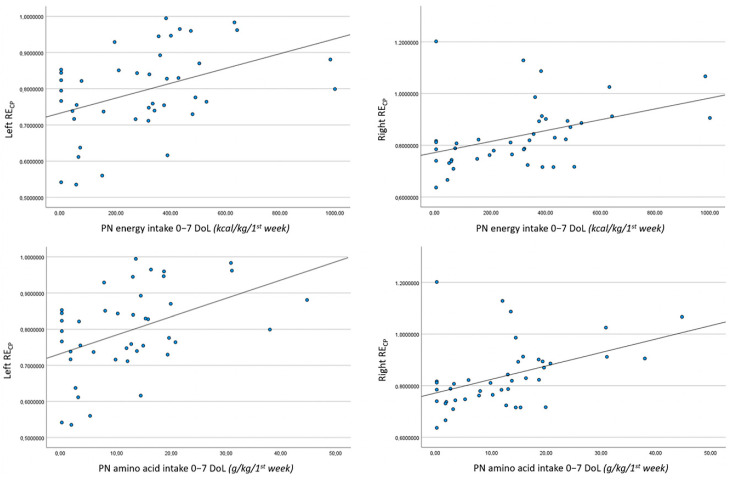
Significant correlations between RE_CP_ values from both left and right parasagittal scans at term-equivalent age and parenteral energy/amino acid intake per kg body weight during the first week of life in preterm newborns. Parenteral energy intake 0–7 DoL (kcal/kg/1st week): left RE_CP_ (*r* = 0.422, *p* = 0.005) and right RE_CP_ (*r* = 0.413, *p* = 0.007). Parenteral amino acid intake 0–7 DoL (g/kg/1st week): left RE_CP_ (*r* = 0.446, *p* = 0.003) and right RE_CP_ (*r* = 0.438, *p* = 0.004).

Correlations between right and left RE_CP_ values at TEA and characteristics of the study population, including intakes of energy and macronutrients by both enteral and parenteral routes in the first week of life, are shown in Table 4. We found a significant negative relationship between BW and right RE_CP_ (r = −0.337, *p* = 0.029). Furthermore, we demonstrated a statistically significant, positive relationship between duration of PN administration and right RE_CP_ (r = 0.335, *p* = 0.030); a similar relation was proved between timing of introduction of EN and left RE_CP_ values (r = 0.308, *p* = 0.048). Notably, energy and protein intakes administered through the parenteral route in the first week of life positively correlated with both right and left RE_CP_ values at TEA (parenteral energy intake vs. right RE_CP_: r = 0.413, *p* = 0.007; parenteral energy intake vs. left RE_CP_: r = 0.422, *p* = 0.005; parenteral amino acid intake vs. right RE_CP_: r = 0.438, *p* = 0.004; parenteral amino acid intake vs. left RE_CP_: r = 0.446, *p* = 0.003). Statistically significant, positive relationships between parenteral intakes of energy and amino acids in the first week of life and RE_CP_ values of both sides at TEA are graphically represented in Figure 2.

The results of multivariate linear regression analysis between right and left RE_CP_ values at TEA and parenteral intakes of energy and amino acids in the first week of life are shown in Table 5 and Table 6.

As regards the relationship between right RE_CP_ at TEA and four predictors (duration of invasive mechanical ventilation (MV), start of EN, duration of PN, and parenteral energy intake in the first week of life), we demonstrated that the overall model was statistically significant (F = 21,008; *p* = 0.000). Thus, the combined predictors significantly explained the variance in right RE_CP_ values. The model accounted for a substantial proportion of the variance in right RE_CP_ values at TEA (R^2^ = 0.689). Among predictors, only parenteral energy intake in the first week of life reached statistical significance (*t* = 3335; *p* = 0.002). The standardized coefficient (β) of this variable suggested that parenteral energy intake in the first week of life had a positive strong impact (β = 0.544) on right RE_CP_ values at TEA.

Regarding the relation between left RE_CP_ at TEA and four predictors, including parenteral energy intake in the first week of life, we found that the overall model was statistically significant (F = 21,135; *p* = 0.000). Thus, the combined predictors significantly explained the variance in left RE_CP_ values. Particularly, the model accounted for a substantial proportion of the variance in left RE_CP_ values at TEA (R^2^ = 0.690). Among predictors, only parenteral energy intake in the first week of life reached statistical significance (*t* = 3450; *p* = 0.001). The standardized coefficient of this variable suggested that parenteral energy intake in the first week of life had a positive strong impact (β = 0.562) on left RE_CP_ values at TEA.

We also investigated the relationship between right RE_CP_ at TEA and four predictors (duration of invasive MV, start of EN, duration of PN, and parenteral amino acid intake in the first week of life). The overall model was statistically significant (F = 19,745; *p* = 0.000), indicating that the combined predictors significantly explained the variance in right RE_CP_ values. In addition, this model accounted for a substantial proportion of the variance in right RE_CP_ values at TEA (R^2^ = 0.675). Among predictors, both parenteral amino acid intake in the first week of life and duration of PN reached statistical significance (*t* = 3015 and *p* = 0.005 for parenteral amino acid intake; *t* = 2218 and *p* = 0.033 for duration of PN). The standardized coefficients of these variables showed that both had a positive impact on right RE_CP_ values at TEA (β = 0.515 for parenteral amino acid intake, β = 0.426 for duration of PN). These results suggested that parenteral amino acid intake in the first week of life and duration of PN are key factors influencing right RE_CP_ values at TEA, with the first of the two having the strongest positive impact.

Finally, regarding the relation between left RE_CP_ and four predictors including parenteral amino acid intake in the first week of life, we found that the overall model was statistically significant (F = 19,764; *p* = 0.000); this indicates that the combined predictors significantly explained the variance in left RE_CP_ values. This model accounted for a substantial proportion of the variance in left RE_CP_ values at TEA (R^2^ = 0.675). However, among predictors, only parenteral amino acid intake in the first week of life showed statistical significance (*t* = 3109; *p* = 0.004). The standardized coefficient of this variable suggested that parenteral amino acid intake in the first week of life had a positive strong impact (β = 0.531) on left RE_CP_ values at TEA.

Post-hoc statistical power for multiple regression studies was 1.0, thus demonstrating that sample size was large enough to detect meaningful results.

ICCs showed high reliability, regarding both inter-observer [right RE_CP_: 0.967 (95% CI = 0.939–0.982); left RE_CP_: 0.973 (95% CI = 0.949–0.985)] and intra-observer [right RE_CP_: 0.960 (95% CI = 0.925–0.978); left RE_CP_: 0.972 (95% CI = 0.948–0.985)] variability in the identification of the highest RE_CP_ value in both sides of the brain.

## 4. Discussion

Quantitative analysis of parieto-occipital periventricular WM echogenicity in preterm-born babies revealed that larger amounts of energy and amino acids early in life were associated with an increased risk of cerebral damage, when administered through the parenteral route.

Although several intervention and observational studies have formerly analyzed the influence of dietary approach on WM development in preterm infants by means of different neuroimaging techniques [2,37,38,39,40,41,42,43,44,45,46,47,48,49,50,51], a large part of these investigations focused exclusively on the effect of cumulative intakes of energy and macronutrients on the developing WM, without considering the route of nutrient administration [37,38,44,45,46,47,48,51]. Given the potential impact of this factor on neurodevelopment and cerebral growth in preterm infants [52], it is essential to evaluate the effects of nutritional strategies, taking into account the route of nutrient administration. Considering the studies analyzing effects of enteral and parenteral intakes separately [2,39,40,41,42,43,49,50], we found that most of these investigations showed a positive impact of feeding strategies in which nutrients were mainly administered through the enteral route since the first days of life [2,39,40,41,43,49].

In this regard, prior studies pointed out a positive association between enteral protein intake and fractional anisotropy (FA) in several WM tracts [39,40,41,49]; additionally, a similar relation was demonstrated for enteral carbohydrate [39], lipid [39,40,41], and energy [39,40,41] intakes. Sato et al. showed that patients receiving higher protein, lipid, and energy intakes through the enteral route early in life reported smaller radial diffusivity (RD) values in the corpus callosum and posterior thalamic radiation, compared to patients receiving lower intakes [41]. Even recent evidence confirms the relation between nutritional strategies in early life and subsequent brain development. As proof, Boscarino et al. observed that enteral energy intake in the first week of life was positively associated with the ultrasonographic growth in length of the corpus callosum during the first 28 postnatal days–together with BW–in preterm-born infants [43]. In support of this hypothesis, we demonstrated that meeting target nutritional intakes too early in life, when the patient is clinically unstable, through parenteral administration of high macronutrient amounts is associated with WM damage identifiable through quantitative analysis of PBI on CUS scans.

Early enteral feeding has the potential to reduce the incidence of some life-challenging conditions–such as necrotizing enterocolitis and late-onset infections–by improving the functional adaptation of the gastrointestinal tract and disrupting patterns of adverse microbial colonization [53,54]. Delayed enteral feeding and intolerance due to intestinal dysmotility increase the time to full enteral feeds and prolong the duration of intravenous nutrition, thus increasing the risk of infection and metabolic complications that may adversely affect brain development [53,55].

In past research, we observed that nerve growth factor (NGF) serum levels at 28 days of life positively correlated with enteral protein and energy intakes in the first week of life, whereas they negatively correlated with parenteral amino acid and energy intake administered during the same time period in VLBW infants [1]. Given the role of NGF in promotion of neuron survival and synaptic remodeling [1], and even in the differentiation of neural stem cells into oligodendrocytes [56], the route of administration of nutrients could actually have an impact on brain development. In addition, data from our research group showed that serum levels of neurofilament light chain (NfL)—a specific and reliable biomarker of cerebral damage [57,58,59]—at 28 days of life were significantly lower in VLBW newborns in which enteral feeding was started within three days of life as compared to patients receiving nutrients through the enteral route for the first time after this time point. We also observed a direct correlation between NfL serum levels on postnatal day 28 and PN duration in both groups (early EN and late EN), thus evidencing that higher concentrations of NfL corresponded to long-lasting PN.

In the present study, we observed an association between longer duration of PN administration and higher parieto-occipital periventricular RE_CP_ values, or occurrence of WM injury in this brain region. Similarly to our studies, some investigations demonstrated that parenteral feeding duration is associated with a higher risk of WM injury expressed as increased WM abnormality MRI score according to the Kidokoro system [60], or reduced FA in several WM tracts [40,61] at TEA [40,60,61]. Notably, Brouwer et al. demonstrated that infants with GA at birth < 28 weeks and PN > 21 days reported significantly higher WM abnormality scores with respect to patients subjected to PN for ≤ 21 days [60]. Coviello et al. enrolled infants with GA < 31 weeks and found that duration of PN was negatively associated with FA in the posterior limb of the internal capsule at TEA MRI [40]. Finally, Barnett et al. recruited newborns with GA at birth < 34 weeks and demonstrated that days of PN were negatively associated with FA in several WM regions at TEA [61].

Even if MRI is the gold-standard method in the field of central nervous system imaging, quantitative analysis of CUS scans enabled us to overcome the issue of discrepancies in technical acquisition of images, and operator-dependent diversity in the interpretation of periventricular hyperechogenicities, thus increasing the diagnostic reliability of brain ultrasonography [4]. Quantitative assessment of echogenicity improves the interpretation of scans and increases the potential of CUS, especially in those settings where MRI is not available or not accessible, or the sonographer is alone during his working activity without possibilities to share his opinions with further colleagues. In addition, this method could permit us to outline a precise trajectory of brain development in preterm-born babies over time. Quantitative analysis of CUS scans has already been used in past research to determine the relationship between WM echogenicity and postnatal and postmenstrual ages, and the relationship between quantitative WM echogenicity and neuromotor development at TEA in a cohort of preterm infants [18]. Particularly, Beller et al. demonstrated that RE_CP_ of the frontoparietal WM decreased significantly with advancing postnatal and postmenstrual ages [18]. Similarly, echogenicity values of frontoparietal and parieto-occipital WM relative to that of calvarium bone at 2–5 weeks postnatal age and during the predischarge study, respectively, were significantly associated with neuromotor status at term [18].

We hypothesize that some mechanisms may explain the observed results. First, parenteral administration of nutrients is associated with a large number of side effects (i.e., hyperglycemia, hyperkalemia, metabolic acidosis, elevated serum blood urea nitrogen, and high plasma ammonia concentrations), which may affect WM development through increased levels of oxidative stress and inflammation [52,62]. Furthermore, preterm birth results in a decreased or postponed expression of antioxidant enzymes in oligodendrocyte precursor cells, which may become even more vulnerable to oxidative injury and undergo necrosis or apoptosis [4,63]. Additionally, mitochondrial impairment in critically ill newborns may exacerbate the production of reactive oxygen species and play a role in the occurrence of cerebral damage following administration of high energy intakes by the parenteral route in these patients [64,65,66,67]. Furthermore, the presence of reactive oxygen species also causes the proliferation of oligodendrocyte precursor cells that are not able to generate myelin [4,63]. Therefore, preterm birth and aggressive PN may be responsible for impaired myelination with subsequent WM damage and structural reorganization [4,63]. A further aspect to consider concerns the gut–brain axis. According to this hypothesis, trophic stimulation of intestinal mucosa by nutrients may promote the secretion of neurotrophins and other molecules in peripherical tissues which control cerebral development [52]. Thus, parenteral administration of high nutrient intakes implies reduced function of the axis with possible brain damage and even impairment of the myelination process [52]. Despite the increasing body of evidence on the mechanisms of brain damage in preterm newborns, further studies are advocated to directly assess the ways in which parenteral administration of nutrients could negatively impact cerebral development in preterm babies.

Despite being interesting, the results of this investigation should be interpreted while taking into account specific limitations. First, the association between early energy and amino acid intake through the parenteral route and RE_CP_ values at TEA may be related to the effects of chance (random error), bias, or confounding factors. However, we verified that the relation with RE_CP_ values persisted even after correction for confounding variables. To limit detection bias, the researchers involved in RE_CP_ measurements were unaware of clinical information. In addition, they entered encrypted records in the dedicated database, and a blinded statistician carried out data analysis in order to minimize the likelihood of assessment bias. Even if no significant variations in NICU practice occurred during the 12-month-long study period, we cannot exclude that some unquantifiable variables (e.g., experience of nurses, composition of medical staff) may have, at least in part, influenced the results of our investigation. We excluded all infants with positive genetic prenatal testing or postnatal diagnosis of genetic syndromes; however, an undefined number of the enrolled newborns may be carriers of unknown variants of genes potentially conditioning WM development and its response to injuries. We think this could not have influenced our results, given that we have no reason to believe that these variants—if present—are not distributed randomly among the enrolled babies. Notably, we have shown a substantial robustness of multivariate analysis; however, the results of our investigation were obtained from a specific population and could change accordingly with characteristics of the study sample. The present research defines the effects of nutritional strategies on brain development up to TEA, a period of life in which the evaluation of neurodevelopment through designated tests is still not reliable. Thus, our study contributes to increase the diagnostic potentiality of CUS, a non-invasive, cost-effective, and highly widespread tool, in early identification of patients at high-risk of neurodevelopmental sequelae.

## 5. Conclusions

Even if MRI is the gold-standard technique for brain imaging, our results encourage the use of quantitative analysis of PBI as a method to improve the interpretation of brain ultrasonography. This technology could be considered a simple, risk-free and repeatable option to investigate the effects of nutrition on WM development. Administration of nutrients should mainly occur through the enteral route from the first week of life, whereas the parenteral route should not be deemed as an alternative to EN for meeting the dietary requirements of preterm newborns. It would be more prudent to reach target nutritional intakes in accordance with the babies’ tolerance to EN rather than achieving them from the first postnatal days through the use of aggressive PN, and monitor the effects of nutritional strategy on the brain through a non-invasive tool. Given the need to promote safety rather than effectiveness in the field of neonatal nutrition, this paper suggests a review of current guidelines [68,69,70] in accordance with recent advances. In addition, it would be interesting to evaluate the long-term neurodevelopment of our patients and assess the relationship between nutritional strategies in early life, brain ultrasonographic appearance at TEA, and long-term neurodevelopment. Further targeted studies are also needed in order to render quantitative evaluations of brain echogenicity through the assessment of mPBI a routinary technique in daily clinical settings.

## Figures and Tables

**Table 1:** Characteristics of the study population (*n* = 42). Data are expressed as mean ± standard deviation, when not specified.

Gestational age, weeks	29.14 ± 2.31
Birth weight, g	1266.60 ± 425.83
Male sex, *n* (%)	17 (40.5)
Cesarean section, *n* (%)	38 (90.5)
Twins, *n* (%)	13 (31.0)
1 min Apgar score	6.38 ± 1.65
5 min Apgar score	8.19 ± 0.89
Arterial cord blood pH	7.29 ± 0.07
Base excess on arterial cord blood	−4.46 ± 2.92
Clinical risk index for babies-II score	6.33 ± 3.92
Small for gestational age, *n* (%)	6 (14.3)
Intrauterine growth restriction, *n* (%)	6 (14.3)
Necrotizing enterocolitis, *n* (%)	3 (7.1)
Intraventricular hemorrhage grade I–II, *n* (%)	0 (0.0)
Periventricular leukomalacia, *n* (%)	16 (38.1)
Sepsis proven by positive cultures, *n* (%)	2 (4.8)
Retinopathy of prematurity, *n* (%)	7 (16.7)
Bronchopulmonary dysplasia, *n* (%)	0 (0.0)
Patent ductus arteriosus, *n* (%)	10 (23.8)
Anemia of prematurity, *n* (%)	10 (23.8)
Duration of invasive mechanical ventilation, days	2.38 ± 1.24
Start of enteral nutrition, days of life	1.33 ± 0.80
Duration of parenteral nutrition, days	14.79 ± 12.76
Length of hospital stay, days	60.17 ± 23.69

**Table 2:** Maternal characteristics of the study population (*n* = 42). Data are expressed as mean ± standard deviation, when not specified.

Maternal age, years	33.38 ± 3.71
Gestational diabetes, *n* (%)	4 (9.5)
Maternal hypertension, *n* (%)	8 (19.0)
Abnormal uterine artery Doppler flow velocimetry, *n* (%)	9 (21.4)
Maternal thyroid disorders during pregnancy, *n* (%)	4 (9.5)
Placental abruption, *n* (%)	4 (9.5)
Antenatal steroids ^a^, *n* (%)	32 (76.2)

^a^ Intramuscular steroids cycle in two doses of 12 mg over a 24 h period.

**Table 3:** Enteral and parenteral nutritional intakes of the study population (*n* = 42). Data are expressed as mean ± standard deviation.

Parenteral energy intake 0–7 DoL (kcal/kg/1st week)	346.96 ± 158.78
Parenteral amino acid intake 0–7 DoL (g/kg/1st week)	15.05 ± 4.50
Parenteral lipid intake 0–7 DoL (g/kg/1st week)	9.96 ± 7.12
Parenteral carbohydrate intake 0–7 DoL (g/kg/1st week)	44.60 ± 16.29
Enteral energy intake 0–7 DoL (kcal/kg/1st week)	191.93 ± 146.86
Enteral protein intake 0–7 DoL (g/kg/1st week)	5.66 ± 4.33
Enteral fat intake 0–7 DoL (g/kg/1st week)	9.67 ± 7.99
Enteral carbohydrate intake 0–7 DoL (g/kg/1st week)	19.13 ± 15.75

Table legend: DoL, day(s) of life.

**Table 4:** Correlations between characteristics of the study population, including enteral and parenteral nutritional intakes, and RE_CP_ values from both left and right parasagittal scans at term-equivalent age.

		Left RE_CP_	Right RE_CP_
Clinical variables	Gestational age (weeks)	r = −0.140	r = −0.179
	Birth weight (g)	r = −0.121	r = −0.337 *
	Arterial cord blood pH	r = 0.148	r = 0.028
	Base excess on arterial cord blood	r = 0.047	r = −0.174
	Clinical risk index for babies-II score	r = 0.034	r = 0.125
	Duration of invasive mechanical ventilation (days)	r = 0.053	r = 0.221
	Start of enteral nutrition (days of life)	r = 0.308 *	r = 0.248
	Duration of parenteral nutrition (days)	r = 0.235	r = 0.335 *
	Length of hospital stay (days)	r = 0.176	r = 0.246
	Maternal age (years)	r = 0.025	r = 0.128
Nutritional variables	Parenteral energy intake 0–7 DoL (kcal/kg/1st week)	r = 0.422 *	r = 0.413 *
	Parenteral amino acid intake 0–7 DoL (g/kg/1st week)	r = 0.446 *	r = 0.438 *
	Parenteral lipid intake 0–7 DoL (g/kg/1st week)	r = 0.306	r = 0.198
	Parenteral carbohydrate intake 0–7 DoL (g/kg/1st week)	r = 0.222	r = 0.264
	Enteral energy intake 0–7 DoL (kcal/kg/1st week)	r = −0.276	r = −0.261
	Enteral protein intake 0–7 DoL (g/kg/1st week)	r = −0.284	r = −0.260
	Enteral fat intake 0–7 DoL (g/kg/1st week)	r = −0.279	r = −0.267
	Enteral carbohydrate intake 0–7 DoL (g/kg/1st week)	r = −0.279	r = −0.260

Table legend: DoL, day(s) of life; RE_CP_, echogenicity of parieto-occipital periventricular white matter relative to that of homolateral choroid plexus; *, statistically significant (*p* < 0.05).

**Table 5:** Multivariate analysis of covariates influencing right RE_CP_ values at 37^0/7^–41^6/7^ weeks postmenstrual age in preterm newborns.

Dependent Variable	Right RE_CP_ at TEA	B	S.E.	β	*p*-Value	95% C.I. for B	
						Lower	Upper
Covariates (model I)	Duration of invasive MV	−0.014	0.013	−0.125	0.279	−0.040	0.012
	Start of EN	0.007	0.058	0.018	0.909	−0.110	0.124
	Duration of PN	0.016	0.008	0.370	0.062	−0.001	0.032
	Parenteral energy intake 0–7 DoL °	0.001	0.000	0.544	0.002 *	0.000	0.002
Covariates (model II)	Duration of invasive MV	−0.015	0.013	−0.137	0.244	−0.042	0.011
	Start of EN	−0.006	0.060	−0.015	0.924	−0.127	0.116
	Duration of PN	0.018	0.008	0.426	0.033 *	0.002	0.034
	Parenteral amino acid intake 0–7 DoL °	0.027	0.009	0.515	0.005 *	0.009	0.046

Legend: C.I., confidence interval; DoL, day(s) of life; EN, enteral nutrition; MV, mechanical ventilation; PN, parenteral nutrition; RE_CP_, echogenicity of parieto-occipital periventricular white matter relative to that of homolateral choroid plexus; S.E., standard error; TEA, term-equivalent age; °, per kg body weight; *, statistically significant (*p* < 0.05).

**Table 6:** Multivariate analysis of covariates influencing left RE_CP_ values at 37^0/7^–41^6/7^ weeks postmenstrual age in preterm newborns.

Dependent Variable	Left RE_CP_ at TEA	B	S.E.	β	*p*-Value	95% C.I. for B	
						Lower	Upper
Covariates (model I)	Duration of invasive MV	−0.017	0.012	−0.158	0.172	−0.041	0.008
	Start of EN	0.023	0.055	0.063	0.682	−0.088	0.134
	Duration of PN	0.013	0.008	0.330	0.095	−0.002	0.029
	Parenteral energy intake 0–7 DoL °	0.001	0.000	0.562	0.001 *	0.000	0.002
Covariates (model II)	Duration of invasive MV	−0.018	0.012	−0.171	0.149	−0.043	0.007
	Start of EN	0.011	0.057	0.029	0.855	−0.105	0.126
	Duration of PN	0.015	0.008	0.387	0.051	0.000	0.031
	Parenteral amino acid intake 0–7 DoL °	0.027	0.009	0.531	0.004 *	0.009	0.044

Legend: C.I., confidence interval; DoL, day(s) of life; EN, enteral nutrition; MV, mechanical ventilation; PN, parenteral nutrition; RE_CP_, echogenicity of parieto-occipital periventricular white matter relative to that of homolateral choroid plexus; S.E., standard error; TEA, term-equivalent age; °, per kg body weight; *, statistically significant (*p* < 0.05).

## Data Availability

The dataset used and analysed during the current study is available from the corresponding author on reasonable request.

## References

[1] De Nardo M.C., Petrella C., Di Chiara M., Di Mario C., Deli G., Travaglia E., Baldini L., Russo A., Parisi P., Fiore M. (2022). Early nutritional intake influences the serum levels of nerve growth factor (NGF) and brain-derived neurotrophic factor in preterm newborns. Front. Neurol..

[2] Isaacs E.B., Gadian D.G., Sabatini S., Chong W.K., Quinn B.T., Fischl B.R., Lucas A. (2008). The Effect of Early Human Diet on Caudate Volumes and IQ. Pediatr. Res..

[3] Terrin G., Boscarino G., Gasparini C., Di Chiara M., Faccioli F., Onestà E., Parisi P., Spalice A., De Nardo M.C., Dito L. (2021). Energy-Enhanced Parenteral Nutrition and Neurodevelopment of Preterm Newborns: A Cohort Study. Nutrition.

[4] Laccetta G., Di Chiara M., De Nardo M.C., Tagliabracci M., Travaglia E., De Santis B., Spiriti C., Dito L., Regoli D., Caravale B. (2023). Quantitative ultrasonographic examination of cerebral white matter by pixel brightness intensity as marker of middle-term neurodevelopment: A prospective observational study. Sci. Rep..

[5] Agut T., Alarcon A., Cabañas F., Bartocci M., Martinez-Biarge M., Horsch S., eurUS.brain group (2020). Preterm white matter injury: Ultrasound diagnosis and classification. Pediatr. Res..

[6] Volpe J.J. (2003). Cerebral White Matter Injury of the Premature Infant—More Common Than You Think. Pediatrics.

[7] Dyet L.E., Kennea N., Counsell S.J., Maalouf E.F., Ajayi-Obe M., Duggan P.J., Harrison M., Allsop J.M., Hajnal J., Herlihy A.H. (2006). Natural History of Brain Lesions in Extremely Preterm Infants Studied with Serial Magnetic Resonance Imaging From Birth and Neurodevelopmental Assessment. Pediatrics.

[8] E Inder T., Wells S.J., Mogridge N.B., Spencer C., Volpe J.J. (2003). Defining the nature of the cerebral abnormalities in the premature infant: A qualitative magnetic resonance imaging study. J. Pediatr..

[9] Horsch S., Hallberg B., Leifsdottir K., Skiöld B., Nagy Z., Mosskin M., Blennow M., Ådén U. (2007). Brain abnormalities in extremely low gestational age infants: A Swedish population based MRI study. Acta Paediatr..

[10] Ancel P.-Y., Livinec F., Larroque B., Marret S., Arnaud C., Pierrat V., Dehan M., N’Guyen S., Escande B., Burguet A. (2006). Cerebral Palsy Among Very Preterm Children in Relation to Gestational Age and Neonatal Ultrasound Abnormalities: The EPIPAGE Cohort Study. Pediatrics.

[11] Martinez-Biarge M., Groenendaal F., Kersbergen K.J., Benders M.J.N.L., Foti F., Cowan F.M., de Vries L.S. (2016). MRI Based Preterm White Matter Injury Classification: The Importance of Sequential Imaging in Determining Severity of Injury. PLoS ONE.

[12] Di Chiara M., Laccetta G., Gangi S., De Santis B., Spiriti C., Attenni M., Bertolaso L., Boscarino G., De Nardo M.C., Ciambra G. (2022). Risk factors and preventive strategies for post-traumatic stress disorder in neonatal intensive care unit. Front. Psychol..

[13] Shankaran S., Laptook A.R., Sood B.G., Do B., Stoll B.J., Van Meurs K.P., Bell E.F., Das A., Barks J., Sarkar S. (2015). Screening Cranial Imaging at Multiple Time Points Improves Cystic Periventricular Leukomalacia Detection. Am. J. Perinatol..

[14] Leijser L.M., de Bruïne F.T., van der Grond J., Steggerda S.J., Walther F.J., van Wezel-Meijler G. (2010). Is sequential cranial ultrasound reliable for detection of white matter injury in very preterm infants?. Neuroradiology.

[15] Skiöld B., Hallberg B., Vollmer B., Ådén U., Blennow M., Horsch S. (2019). A Novel Scoring System for Term-Equivalent-Age Cranial Ultrasound in Extremely Preterm Infants. Ultrasound Med. Biol..

[16] Kuban K., Adler I., Allred E.N., Batton D., Bezinque S., Betz B.W., Cavenagh E., Durfee S., Ecklund K., Feinstein K. (2007). Observer variability assessing US scans of the preterm brain: The ELGAN study. Pediatr. Radiol..

[17] Harris D.L., Bloomfield F.H., Teele R.L., E Harding J., Australian and New Zealand Neonatal Network (2006). Variable interpretation of ultrasonograms may contribute to variation in the reported incidence of white matter damage between newborn intensive care units in New Zealand. Arch. Dis. Child. Fetal Neonatal Ed..

[18] Beller T., Peylan T., Ben Sira L., Shiran S.I., Levi L., Bassan H. (2016). Quantitative analysis of cranial ultrasonographic periventricular echogenicity in relation to early neuromotor development in preterm infants. Arch. Dis. Child. Fetal Neonatal Ed..

[19] Padilla N.F., Enriquez G., Jansson T., Gratacos E., Hernandez-Andrade E. (2009). Quantitative Tissue Echogenicity of the Neonatal Brain Assessed by Ultrasound Imaging. Ultrasound Med. Biol..

[20] Pinto P.S., Tekes A., Singhi S., Northington F.J., Parkinson C., Huisman T.A.G.M. (2012). White–gray matter echogenicity ratio and resistive index: Sonographic bedside markers of cerebral hypoxic–ischemic injury/edema?. J. Perinatol..

[21] Simaeys B., Philips W., Lemahieu I., Govaert P. (2000). Quantitative analysis of the neonatal brain by ultrasound. Comput. Med. Imaging Graph..

[22] Ichihashi K., Yada Y., Takahashi N., Homma Y., Momoi M. (2008). Integrated backscatter of the brain of preterm infants. J. Perinat. Med..

[23] Fujimoto C., Yamashita Y., Kanda H., Harada E., Maeno Y., Matsuishi T. (2003). In vivo quantitative ultrasonic evaluation of neonatal brain with a real time integrated backscatter imaging system. Brain Dev..

[24] Hope T., Gregson P., Linney N., Schmidt M. (2004). Ultrasonic Tissue Characterization as a Predictor of White Matter Damage: Results of a Preliminary Study. IEEE Ultrasonics Symposium.

[25] Na Jung H., Suh S.-I., Park A., Kim G.-H., Ryoo I. (2019). Early Prediction of Periventricular Leukomalacia Using Quantitative Texture Analysis of Serial Cranial Ultrasound Scans in Very Preterm Infants. Ultrasound Med. Biol..

[26] Narchi H., Mahmoud-Ghoneim D., Skinner A., Cogings P. (2013). Texture analysis of periventricular echogenicity on neonatal cranial ultrasound predicts periventricular leukomalacia. J. Neonatal-Perinat. Med..

[27] Tenorio V., Bonet-Carne E., Botet F., Marques F., Amat-Roldan I., Gratacos E. (2011). Correlation Between a Semiautomated Method Based on Ultrasound Texture Analysis and Standard Ultrasound Diagnosis Using White Matter Damage in Preterm Neonates as a Model. J. Ultrasound Med..

[28] You S.K., Choi Y.H., Park S.J., Cheon J.-E., Kim I.-O., Kim W.-S., Lee S.M., Cho H.-H. (2015). Quantitative Sonographic Texture Analysis in Preterm Neonates with White Matter Injury: Correlation of Texture Features with White Matter Injury Severity. J. Ultrasound Med..

[29] Broderick P.A. (2022). A New Approach to Tumor Cancer with a Novel Imaging Profile for White matter abnormalities including Leukodystrophies: Sensing the Human Brain. Med. Res. Arch..

[30] Lal B.K., Hobson R.W., Pappas P.J., Kubicka R., Hameed M., Chakhtura E.Y., Jamil Z., Padberg F.T., Haser P.B., Durán W.N. (2002). Pixel distribution analysis of B-mode ultrasound scan images predicts histologic features of atherosclerotic carotid plaques. J. Vasc. Surg..

[31] American Academy of Pediatrics Committee on Fetus and Newborn (2012). Levels of neonatal care. Pediatrics.

[32] De Vries L.S., Eken P., Dubowitz L.M. (1992). The spectrum of leukomalacia using cranial ultrasound. Behav. Brain Res..

[33] Laccetta G., Fiori S., Giampietri M., Ferrari A., Cetica V., Bernardini M., Chesi F., Mazzotti S., Parrini E., Ciantelli M. (2019). A de novo KCNQ2 Gene Mutation Associated with Non-familial Early Onset Seizures: Case Report and Revision of Literature Data. Front. Pediatr..

[34] Terrin G., Passariello A., Canani R.B., Manguso F., Paludetto R., Cascioli C. (2009). Minimal enteral feeding reduces the risk of sepsis in feed-intolerant very low birth weight newborns. Acta Paediatr..

[35] Terrin G., Stronati L., Cucchiara S., De Curtis M. (2017). Serum Markers of Necrotizing Enterocolitis: A Systematic Review. J. Pediatr. Gastroenterol. Nutr..

[36] Anvari A., Halpern E.F., Samir A.E. (2018). Essentials of Statistical Methods for Assessing Reliability and Agreement in Quantitative Imaging. Acad. Radiol..

[37] Janson E., Willemsen M.F., Van Beek P.E., Dudink J., Van Elburg R.M., Hortensius L.M., Tam E.W.Y., de Pipaon M.S., Lapillonne A., de Theije C.G.M. (2023). The influence of nutrition on white matter development in preterm infants: A scoping review. Pediatr. Res..

[38] Strømmen K., Blakstad E.W., Moltu S.J., Almaas A.N., Westerberg A.C., Amlien I.K., Rønnestad A.E., Nakstad B., Drevon C.A., Bjørnerud A. (2014). Enhanced Nutrient Supply to Very Low Birth Weight Infants is Associated with Improved White Matter Maturation and Head Growth. Neonatology.

[39] Schneider J., Fumeaux C.J.F., Duerden E.G., Guo T., Foong J., Graz M.B., Hagmann P., Chakravarty M.M., Hüppi P.S., Beauport L. (2018). Nutrient Intake in the First Two Weeks of Life and Brain Growth in Preterm Neonates. Pediatrics.

[40] Coviello C., Keunen K., Kersbergen K.J., Groenendaal F., Leemans A., Peels B., Isgum I., Viergever M.A., De Vries L.S., Buonocore G. (2018). Effects of early nutrition and growth on brain volumes, white matter microstructure, and neurodevelopmental outcome in preterm newborns. Pediatr. Res..

[41] Sato J., Vandewouw M.M., Bando N., Ng D.V.Y., Branson H.M., O’connor D.L., Unger S.L., Taylor M.J. (2021). Early nutrition and white matter microstructure in children born very low birth weight. Brain Commun..

[42] Terrin G., De Nardo M.C., Boscarino G., Di Chiara M., Cellitti R., Ciccarelli S., Gasparini C., Parisi P., Urna M., Ronchi B. (2020). Early Protein Intake Influences Neonatal Brain Measurements in Preterms: An Observational Study. Front. Neurol..

[43] Boscarino G., Di Chiara M., Cellitti R., De Nardo M.C., Conti M.G., Parisi P., Spalice A., Di Mario C., Ronchi B., Russo A. (2021). Effects of early energy intake on neonatal cerebral growth of preterm newborn: An observational study. Sci. Rep..

[44] Ottolini K.M., Andescavage N., Kapse K., Jacobs M., Murnick J., Veer R.V., Basu S., Said M., Limperopoulos C. (2021). Early Lipid Intake Improves Cerebellar Growth in Very Low-Birth-Weight Preterm Infants. J. Parenter. Enter. Nutr..

[45] Power V.A., Spittle A.J., Lee K.J., Anderson P.J., Thompson D.K., Doyle L.W., Cheong J.L. (2019). Nutrition, Growth, Brain Volume, and Neurodevelopment in Very Preterm Children. J. Pediatr..

[46] Hansen-Pupp I., Hövel H., Hellström A., Hellström-Westas L., Löfqvist C., Larsson E.-M., Lazeyras F., Fellman V., Hüppi P.S., Ley D. (2011). Postnatal decrease in circulating insulin-like growth factor-I and low brain volumes in very preterm infants. J. Clin. Endocrinol. Metab..

[47] van Beek P.E., Claessens N.H., Makropoulos A., Groenendaal F., de Vries L.S., Counsell S.J., Benders M.J. (2020). Increase in Brain Volumes after Implementation of a Nutrition Regimen in Infants Born Extremely Preterm. J. Pediatr..

[48] Rozé J.-C., Morel B., Lapillonne A., Marret S., Guellec I., Darmaun D., Bednarek N., Moyon T., Marchand-Martin L., Benhammou V. (2021). Association Between Early Amino Acid Intake and Full-Scale IQ at Age 5 Years Among Infants Born at Less Than 30 Weeks’ Gestation. JAMA Netw. Open.

[49] Hortensius L.M., Janson E., van Beek P.E., Groenendaal F., Claessens N.H.P., de Veye H.F.N.S., Eijsermans M.J.C., Koopman-Esseboom C., Dudink J., van Elburg R.M. (2021). Nutritional Intake, White Matter Integrity, and Neurodevelopment in Extremely Preterm Born Infants. Nutrients.

[50] Beauport L., Schneider J., Faouzi M., Hagmann P., Hüppi P.S., Tolsa J.-F., Truttmann A.C., Fumeaux C.J.F. (2017). Impact of Early Nutritional Intake on Preterm Brain: A Magnetic Resonance Imaging Study. J. Pediatr..

[51] Tan M., Abernethy L., Cooke R. (2008). Improving head growth in preterm infants—A randomised controlled trial II: MRI and developmental outcomes in the first year. Arch. Dis. Child. Fetal Neonatal Ed..

[52] De Nardo M.C., Di Mario C., Laccetta G., Boscarino G., Terrin G. (2022). Enteral and parenteral energy intake and neurodevelopment in preterm infants: A systematic review. Nutrition.

[53] Cormack B.E., Harding J.E., Miller S.P., Bloomfield F.H. (2019). The Influence of Early Nutrition on Brain Growth and Neurodevelopment in Extremely Preterm Babies: A Narrative Review. Nutrients.

[54] Berrington J.E., Stewart C.J., Embleton N.D., Cummings S.P. (2013). Gut microbiota in preterm infants: Assessment and relevance to health and disease. Arch. Dis. Child. Fetal Neonatal Ed..

[55] Rønnestad A., Abrahamsen T.G., Medbø S., Reigstad H., Lossius K., Kaaresen P.I., Egeland T., Engelund I.E., Irgens L.M., Markestad T. (2005). Late-Onset Septicemia in a Norwegian National Cohort of Extremely Premature Infants Receiving Very Early Full Human Milk Feeding. Pediatrics.

[56] Langhnoja J., Buch L., Pillai P. (2021). Potential role of NGF, BDNF, and their receptors in oligodendrocytes differentiation from neural stem cell: An in vitro study. Cell Biol. Int..

[57] Shah D.K., Yip P.K., Barlas A., Tharmapoopathy P., Ponnusamy V., Michael-Titus A.T., Chisholm P. (2020). Raised Plasma Neurofilament Light Protein Levels After Rewarming Are Associated with Adverse Neurodevelopmental Outcomes in Newborns After Therapeutic Hypothermia. Front. Neurol..

[58] Abdelhak A., Petermeier F., Benkert P., Schädelin S., Oechtering J., Maceski A.M., Kabesch M., Geis T., Laub O., Leipold G. (2023). Serum neurofilament light chain reference database for individual application in paediatric care: A retrospective modelling and validation study. Lancet Neurol..

[59] Schultz S.A., Strain J.F., Adedokun A., Wang Q., Preische O., Kuhle J., Flores S., Keefe S., Dincer A., Ances B.M. (2020). Serum neurofilament light chain levels are associated with white matter integrity in autosomal dominant Alzheimer’s disease. Neurobiol. Dis..

[60] Brouwer M.J., Kersbergen K.J., van Kooij B.J.M., Benders M.J.N.L., van Haastert I.C., Koopman-Esseboom C., Neil J.J., de Vries L.S., Kidokoro H., Inder T.E. (2017). Preterm brain injury on term-equivalent age MRI in relation to perinatal factors and neurodevelopmental outcome at two years. PLoS ONE.

[61] Barnett M.L., Tusor N., Ball G., Chew A., Falconer S., Aljabar P., Kimpton J.A., Kennea N., Rutherford M., Edwards A.D. (2017). Exploring the multiple-hit hypothesis of preterm white matter damage using diffusion MRI. NeuroImage Clin..

[62] Boscarino G., Conti M.G., Gasparini C., Onestà E., Faccioli F., Dito L., Regoli D., Spalice A., Parisi P., Terrin G. (2021). Neonatal Hyperglycemia Related to Parenteral Nutrition Affects Long-Term Neurodevelopment in Preterm Newborn: A Prospective Cohort Study. Nutrients.

[63] Cainelli E., Arrigoni F., Vedovelli L. (2020). White matter injury and neurodevelopmental disabilities: A cross-disease (dis)connection. Prog. Neurobiol..

[64] Sánchez-Alvarez R., Almeida A., Medina J.M. (2002). Oxidative Stress in Preterm Rat Brain Is Due to Mitochondrial Dysfunction. Pediatr. Res..

[65] Bale G., Mitra S., de Roever I., Sokolska M., Price D., Bainbridge A., Gunny R., Uria-Avellanal C., Kendall G.S., Meek J. (2019). Oxygen dependency of mitochondrial metabolism indicates outcome of newborn brain injury. J. Cereb. Blood Flow Metab..

[66] McClave S.A., Wischmeyer P.E., Miller K.R., van Zanten A.R.H. (2019). Mitochondrial Dysfunction in Critical Illness: Implications for Nutritional Therapy. Curr. Nutr. Rep..

[67] Casaer M.P., Mesotten D., Hermans G., Wouters P.J., Schetz M., Meyfroidt G., Van Cromphaut S., Ingels C., Meersseman P., Muller J. (2011). Early versus Late Parenteral Nutrition in Critically Ill Adults. New Engl. J. Med..

[68] Terrin G., Boscarino G., Di Chiara M., Iacobelli S., Faccioli F., Greco C., Onestà E., Sabatini G., Pietravalle A., Oliva S. (2020). Nutritional Intake Influences Zinc Levels in Preterm Newborns: An Observational Study. Nutrients.

[69] Joosten K., Embleton N., Yan W., Senterre T., the ESPGHAN/ESPEN/ESPR/CSPEN working group on pediatric parenteral nutrition (2018). ESPGHAN/ESPEN/ESPR/CSPEN guidelines on pediatric parenteral nutrition: Energy. Clin. Nutr..

[70] Van Goudoever J.B., Carnielli V., Darmaun D., de Pipaon M.S., the ESPGHAN/ESPEN/ESPR/CSPEN working group on pediatric parenteral nutrition (2018). ESPGHAN/ESPEN/ESPR/CSPEN guidelines on pediatric parenteral nutrition: Amino acids. Clin. Nutr..

